# *Dehalococcoides mccartyi* strain NIT01 grows more stably in vessels made of pure titanium rather than the stainless alloy SUS304

**DOI:** 10.1111/1758-2229.13192

**Published:** 2023-08-18

**Authors:** Masaki Asai, Yuki Morita, Lingyu Meng, Hidetoshi Miyazaki, Naoko Yoshida

**Affiliations:** ^1^ Department of Civil Engineering Nagoya Institute of Technology Nagoya Japan; ^2^ Department of Physical Science and Engineering Nagoya Institute of Technology Nagoya Japan

## Abstract

Advances in many isolation studies have revealed that pure *Dehalococcoides* grow stably, although the large‐scale pure cultivation of *Dehalococcoides* has yet to be established. In this study, 7 L‐culturing of *Dehalococcoides mccartyi* strain NIT01 was first performed using vessels made of glass and stainless alloy SUS304. All batches cultured in the glass vessel successfully dechlorinated >95% of 1 mM trichloroethene (TCE) to ethene (ETH), whereas only 5 out of 13 batches cultured in the SUS304 vessel did the same. The difference in dechlorination efficiency suggested the possible inhibition of dechlorination by SUS304. Also, the strain NIT01 showed long delays in dechlorination with pieces of SUS316, steel, and a repeatedly used SUS304, but not with titanium. The repeatedly used SUS304 cracked and increased the Fe^2+^ concentration to ≥76 μM. Dechlorination by this strain was also inhibited with ≥1000 μM Fe^2+^ and ≥23 μM Cr^3+^ but not with ≤100 μM Ni^2+^, suggesting that Cr^3+^ eluted from solid stainless alloys inhibited the dechlorination. Culturing in a titanium vessel instead of a stainless alloy showed the complete dechlorination of 1 mM TCE within 12–28 days with a growth yield of 2.7 × 10^7^ cells/μmol‐released Cl^−^, even after repeating use of the vessels six times.

## Graphical abstract

To establish large‐scale cultivation of *Dehalococcoides*, 7–10 litre culturing of *Dehalococcoides mccartyi* strain NIT01 was performed using three different vessels made of glass, stainless alloy SUS304, and titanium. The unbreakable titanium vessel demonstrated complete dechlorination of trichloroethene (TCE) to ethene, even after being reused five times. However, the SUS304 vessels that were repeatedly used showed a substantial delay in the dechlorination of TCE by *Dehalococcoides*.

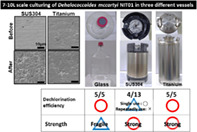

## INTRODUCTION

Although groundwater quality has deteriorated due to agricultural and industrial exploitation (UNESCO, 2021), it is the main source of water for over 2.5 billion people globally (Guppy et al., 2018). One of the most serious concerns regarding groundwater use is the frequent detection of carcinogenic contaminants, such as chloroethene (CE), trichloroethene (TCE), and vinyl chloride (VC), which are listed in the Substance Priority List of the Agency for Toxic Substances and Disease Registry (ATSDR, 2019). This is especially true for groundwater that has been subject to improper disposal procedures and accidental spills from industrial factories that use these chemicals as solvents for cleaning and degreasing.

*Dehalococcoides* is an organohalide‐respiring bacterium that grows via respiration using organohalides as terminal electron acceptors in a membrane‐bound electron transport chain (Adrian & Löffler, 2016; Mohn & Kennedy, 1992). *Dehalococcoides* can dechlorinate CEs to nontoxic ethene (ETH) (Löffler et al., 2013) and has been used extensively in the bioremediation of CE‐contaminated environments (Sun et al., 2000). Several commercially developed consortia that include *Dehalococcoides*, such as KB‐1 culture, have been successfully applied to the bioremediation of CE‐contaminated groundwater (Major et al., 2002; Pérez‐de‐Mora et al., 2018). The purity or complexity of these cultures affects groundwater microbial communities. For example, the inoculation of waste‐activated sludge increases dechlorination but drastically alters the microbial community structure (Lu et al., 2021; Xu et al., 2021), whereas inoculation using a pure *Dehalococcoides* culture has less of a structural effect (Xu et al., 2022). Nutrient minerals and e^−^ donors also affect the microbial community. The use of a C1 e^−^ donor, such as formate, can minimize the anthropogenic disturbance of groundwater ecologies in combination with bioaugmentation using pure *Dehalococcoides* (Tomita et al., 2022). Of the various *Dehalococcoides* strains that have been successfully isolated, a few have shown rapid dechlorination of TCE (0.5–4.0 mM) to ETH within 10–45 days (Asai et al., 2021; Zhao & He, 2018). Furthermore, to the best of our knowledge, the maximum scale of the *Dehalococcoides* pure culture is 7 L for the strain NIT01 in batch mode (Asai et al., 2021) and 1 L for the strain CBDB1 in continuous mode (Reino et al., 2023). The other *Dehalococcoides* strains, such as FL2, 195, and CG1, have been cultured at <1 L (He et al., 2007; Wang et al., 2014; Yan et al., 2021), and the large‐scale culture of pure *Dehalococcoides* has not been commercially available.

So far, several consortia with *Dehalococcoides* have been scaled up to 6–3200 L using stainless alloy vessels for bioaugmentation, although the type of the stainless alloy has not been stated in previous reports. KB‐1 culture has been scaled up to 6 L (Major et al., 2002) and 100 L (Pérez‐de‐Mora et al., 2018), and the consortium SDC‐90 has been scaled up to 3200 L using stainless alloy vessels (Vainberg et al., 2009). KB‐1 culture has been routinely grown by feeding with 0.3 mM TCE and 1.5 mM methanol every 2 weeks (Major et al., 2002), and SDC‐90 has been grown with tetrachloroethene (PCE) continuously fed at with 20–30 μL/h/L culture (corresponding to 0.2–0.3 μmol/h/L; the accumulative concentration of PCE was calculated to be ~1.0–2.0 mmol/L for 35 days of incubation). However, Fe^2+^, Cr^3+^, and Ni^2+^ have possibly been eluted from the stainless alloy (Hedberg et al., 2013) and have been reported to inhibit reductive dechlorination by *Dehalococcoides* at different concentrations. Cr^3+^ at a concentration of ≥38 μM inhibits PCE dechlorination by *Dehalococcoides mccartyi* CG1 (Lu et al., 2020). Fe^2+^ at a concentration of ≥1.75 mM, eluted from ferrihydrite, inhibits TCE dechlorination by the consortium KB‐1 (Paul & Smolders, 2014). Ni^2+^ at a concentration of ≥75 μM inhibits the Ni–Fe hydrogenase activity of *D. mccartyi* strain CBDB1 (Jayachandran et al., 2004). The inhibition of the reductive dechlorination by metal ions has also been reported for As^3+^, As^5+^, Cu^2+^, Cd^2+^, and Pb^2+^ (Gushgari‐Doyle & Alvarez‐Cohen, 2020; Lu et al., 2020). Multiple mechanisms have been described for the inhibition of microbial metabolism caused by metals, including the replacement of essential metals in a metalloprotein by a metal with a higher affinity for the protein (mismetallation; Bennett & Gralnick, 2019; MacOmber & Hausinger, 2011). Twenty‐five percent of all proteins are considered to be metalloproteins (Maret, 2010), and the coordination chemistry of metalloproteins (McCall & Fierke, 2004) the Irving–Williams series (Irving & Williams, 1948) shows that the divalent metal ions interact with ligands in the following order of affinity: Mn^2+^ < Fe^2+^ < Co^2+^ < Ni^2+^ < Cu^2+^ > Zn^2+^. Other mechanisms include the binding of metal ions to catalytic residues of non‐metalloenzymes, the allosteric inhibition of enzymes (MacOmber & Hausinger, 2011), the destruction of iron–sulfur clusters by Cu^+^ (Bennett & Gralnick, 2019; Macomber & Imlay, 2009), and the generation of reactive oxygen species by increasing intra‐cellular iron concentration under aerobic conditions (ferroptosis) (Shen & Naqvi, 2021).

*Dehalococcoides mccartyi* strain NIT01 was isolated from river sediment contaminated with CEs (Ismaeil et al., 2017) and was shown to dechlorinate ≤4 mM TCE of the initial concentration (Asai et al., 2021). This strain can be applied to TCE‐to‐ETH dechlorination driven by an electrode (Meng et al., 2022) and formate (Tomita et al., 2022) in soil and groundwater microcosms. However, the large‐scale culturing of *D. mccartyi* strain NIT01 using a metal vessel has been an obstacle in employing the strain for bioaugmentation.

In this study, we compared TCE‐to‐ETH dechlorination between a *D. mccartyi* strain NIT01 that was batch‐cultured using glass and one that used stainless alloy vessels; this was performed at a scale of 7 L. We also investigated the effect of solid metal pieces and metal ions on dechlorination. Based on our findings, a titanium vessel was proposed as a durable vessel that can aid the efficient growth of *Dehalococcoides* strains.

## MATERIALS AND METHODS

### Culturing of *D. mccartyi* NIT01

*Dehalococcoides mccartyi* NIT01 was routinely cultured using the mineral bromide salt with 30 mM bicarbonate‐buffered medium (DHB‐CO_3_‐Br medium) (Tomita et al., 2022) supplemented with 5 mM acetate as a carbon source, 0.15 mM Na_2_S and 1 mM Ti (III)‐NTA as reducing agents, a 1% (v/v) vitamin mix solution, 0.5% (v/v) or 10 mg/L vitamin B_12_, 80% H_2_ as an electron donor in the headspace gas, and filter‐sterilized 1 mM TCE as an electron acceptor. The DHB‐CO_3_‐Br medium was preliminarily deoxygenated by bubbling with an anaerobic mixed gas (N_2_:CO_2_ = 4:1) and autoclaved in a glass vial sealed with a butyl rubber cap. Further, the headspace was replaced with a filter‐sterilized anaerobic gas mixture (H_2_:CO_2_ = 4:1) and supplemented with acetate, reducing agents, vitamins, and TCE. All cultures were statically incubated at 28°C.

### Large‐scale culturing of *D. mccartyi* strain NIT01

The large‐scale culturing of *D. mccartyi* strain NIT01 was performed using three different types of vessels: an 11.4 L‐SUS304 vessel (FUJITECHNO Co., Ltd., Tokyo, Japan), a 10 L‐glass vessel (FB‐800‐10000, Wexer Lifescience Co., Ltd., Tokyo, Japan), and an 18 L‐titanium vessel (Saito Industry Co., Ltd., Aichi, Japan), wherein the working volumes were 7, 7, and 10 L, respectively. First, the DHB‐CO_3_‐Br medium was autoclaved in closed vessels made of SUS304 and titanium. After covering the glass vessel with aluminium foil, its medium was autoclaved before bubbling the media with the N_2_‐CO_2_ gas mixture to avoid breakage of the vessel. The headspace was then replaced with the H_2_‐CO_2_ gas mixture gas and supplemented with acetate, reducing agents, and vitamins. To inoculate *D. mccartyi* strain NIT01, 0.5–1.6 L of the pre‐culture was first placed in a glass vial and connected to the closed vessel via a needle and a silicon tube, and then injected into the large‐scale vessels by adding the H_2_‐CO_2_ gas mixture to the glass vial. The inoculum rate for culturing in the glass and SUS304 vessels was 19%, and that in the titanium vessel was 5% or 16% (v/v) (Figure S1). Finally, TCE was added at a concentration of 0.24–3.0 mM to the vessel. *D. mccartyi* strain NIT01 was cultured using the large‐scale vessels at least four times.

### Effects of solid metals and ions on dechlorination

To select a metal vessel for the large‐scale culture of *D. mccartyi* strain NIT01, the strain NIT01 was also cultured with pieces of four different metals: SUS304, SUS316, steel (IWATA MFG. Co., Ltd., Gifu, Japan), and pure titanium (KENIS Ltd., Osaka, Japan). Both SUS304 and SUS316 mainly consisted of Fe but differed in their minor components; SUS304 included Cr (18%–20%), Ni (8%–10.5%), S (≤0.030%), P (≤0.045%), Mn (≤2.0%), Si (≤1.0%), and C (≤0.08%), whereas SUS316 included Cr (16%–18%), Ni (10%–14%), Mo (2%–3%), S (≤0.030%), P (≤0.045%), Mn (≤2.0%), Si (≤1.0%), and C (≤0.08%). The purity of the titanium was >99.5%, corresponding to Japanese Industrial Standards Class 2 and American Society for Testing Materials Grade 2. Subsequently, dechlorination by these cultures was compared with dechlorination by cultures without metal pieces (positive control [PC]). Four metal sheets (7.5 mm × 7.5 mm × 0.8 mm) were subjected to ultrasonic washing for 15 min before they were added to fresh medium (20 mL), as described above, in 60‐mL glass bottles, where the ratio of the metal surface to the liquid culture corresponded to that in the large‐scale metal vessels. After autoclaving the medium, which included the metal pieces, we inoculated 5% of the *D. mccartyi* strain NIT01 pre‐culture to the medium, followed by stationary incubation. Upon the complete dechlorination of TCE to ETH within 30 days, the metal pieces were collected, washed, and reused to evaluate the effect of reuse on dechlorination by this strain.

The effect of major metal ions present in the stainless alloy, such as Fe^2+^, Ni^2+^, and Cr^3+^, on dechlorination by *D. mccartyi* strain NIT01 was determined by comparing dechlorination by cultures containing metal salts at different concentrations: FeCl_2_·4H_2_O (273, 100, and 5000 μM), NiCl_2_·6H_2_O (10, 50, and 100 μM), and CrCl_3_·6H_2_O (23, 72, and 500 μΜ).

### Scanning electron microscopy imaging

Scanning electron microscopy (SEM) was used to observe differences in the surfaces of the metal pieces (SUS304, SUS316, steel, and titanium) before and after incubation for 101–192 days. Furthermore, as a control test, metal pieces were also observed before and after culturing without the strain NIT01 for 28 days. The metal pieces were subjected to ultrasonic washing for 15 min to remove dirt on the surface before being photographed with SEM (JSM‐7800F, JEOL Ltd., Tokyo, Japan) operating at 5.0 kV. Surface morphologies were compared among the samples before and after incubation, with and without cells.

### X‐ray fluorescence analysis

X‐ray fluorescence (XRF) mapping was performed using the XGT‐7200V analytical microscope (Horiba Ltd, Kyoto, Japan). Six‐hundred microliters of the culture supplemented with and without metal pieces were placed on a glass slide and dried. The dried slide was analysed by XRF at a current of 1 mA and voltage of 50 keV; the beam size on the sample was 10 μm.

### Analytical methods

CEs and ETH were detected and quantified by gas chromatography (Flame Ionization Detector; Shimadzu, Kyoto, Japan) as described previously (Yoshida et al., 2007). Cell density was determined by direct microscopic counting. The cultures were diluted at a 100–1000 dilution rate with a 10 mM phosphate‐buffered saline solution (pH 7.0) containing 1 mM ethylenediaminetetraacetic acid and stained with 1000‐times diluted SYBR Green I. The cells in the diluted solutions were captured on a black polyvinylidene fluoride membrane filter (0.02 μm pore size, MILLIPORE, Toyo Roshi Kaisha, Ltd., Tokyo, Japan) via suction filtration, dried in the dark, and mounted with glycerol (>99%). The number of stained cells on the membrane filter was counted directly using a fluorescence microscope (Yoshida et al., 2006). The concentration of Fe^2+^ in the cultures was determined by a colorimetric assay using 1,10‐phenanthroline hydrochloride. Two‐and‐a‐half millilitres of the filter‐sterilized sample was mixed in a glove box filled with N_2_ with both 0.5 mL of MilliQ‐water and 2 mL of 2.0 mM 1,10‐phenanthroline hydrochloride monohydrate dissolved in a 20 mM acetic acid buffer (pH 4.7). The absorbance of the prepared sample was measured at a wavelength of 510 nm with a spectrophotometer (NanoPhotometer® P‐Class P330; Implen, Germany). The total concentration of Fe^3+^ and Fe^2+^ in the culture was determined after reduction by adding 0.5 mL of 1.4 M hydroxylammonium chloride instead of MilliQ‐water, and the Fe^3+^ concentration was calculated as a difference in concentration between the total Fe and Fe^2+^.

## RESULTS AND DISCUSSION

### TCE dechlorination by *D. mccartyi* strain NIT01 in stainless alloy and glass vessels

**FIGURE 1:**
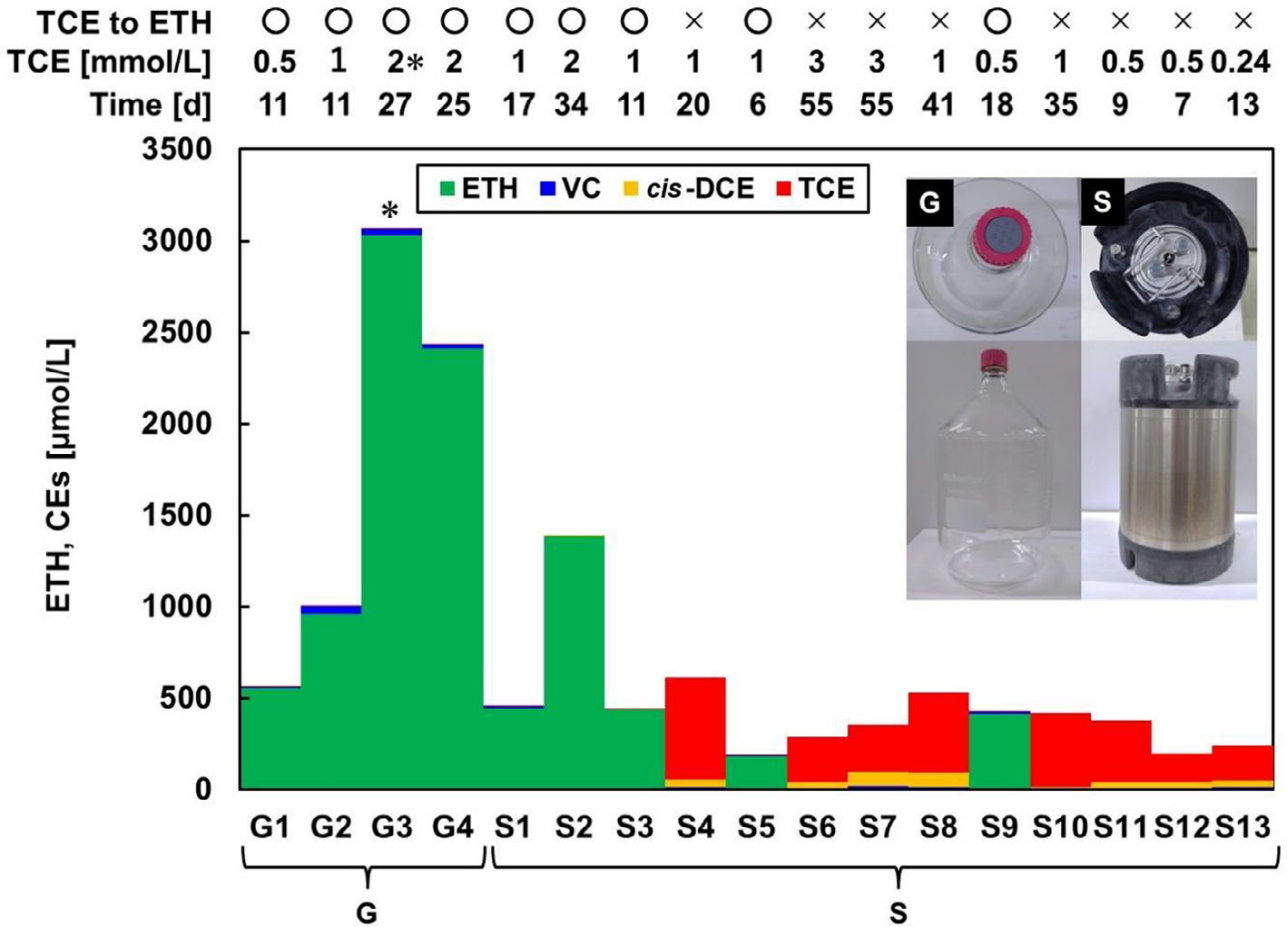
Concentrations of ETH and CEs in a large‐scale culture of *Dehalococcoides mccartyi* strain NIT01 using glass and stainless alloy vessels. G and S indicate glass and SUS304, respectively. Each culture was independently prepared, and the inoculum was cultured at <2 weeks after completing the dechlorination. The TCE concentration is the added concentration. The time (d) indicates the number of days to achieve the respective CEs and ETH concentrations. In addition, the threshold for completion of the dechlorination was set at 95% dechlorination of TCE to ETH. O indicates that dechlorination was completed, and × indicates that dechlorination was interrupted. CEs, chloroethenes; *cis*‐DCE, *cis*‐1,2‐dichloroethene; ETH, ethene; TCE, trichloroethene; VC, vinyl chloride. * denotes that imbalance in the added TCE and detected ETH in G3 is unknown, but was possibly caused by a mistake in the TCE addition. All data in Figure 1 are single determinants from each culture.

The 7 L‐scale culturing of *D. mccartyi* strain NIT01 using glass vessels (capacity: 10 L) stably dechlorinated 95% of 0.5, 1, and 2 mM TCE to ETH within 11, 11, and 27 days, respectively (Figure 1); this occurred in all four glass vessels, Glass (G)1–G4. However, batch culturing 13 times using the stainless alloy SUS304 vessel showed a tendency for the dechlorination to become unstable with repeated use. The first three batches successfully dechlorinated 1 and 2 mM TCE to ETH within 17 and 34 days, respectively; moreover, the time required for TCE‐to‐ETH dechlorination was comparable to that observed for cultures grown using a glass vessel. However, in the subsequent eight batches that reused the vessels, >70% of the spiked TCE remained dechlorinated (Figure 1). Batches SUS304 (S)1–S3, S5, and S9 dechlorinated 95% of 0.5–2 mM TCE to ETH within 34 days. However, batches S4, S6–S8, and S10–S13 spiked with 0.24–3 mM TCE and retained most CEs after 7–55 days of incubation. A total of four stainless alloy vessels were used for the experiments, and the number of uses was not recorded. The reason why batch S9 succeeded is unknown, but it is possible that batch S9 used a new vessel. Collectively, these results suggested that the repeated use of the stainless alloy vessels resulted in the inhibition of *D. mccartyi* strain NIT01‐mediated TCE dechlorination. This is in contrast to the successful large‐scale culturing of *Dehalococcoides* consortia using stainless alloy vessels (Pérez‐de‐Mora et al., 2018; Vainberg et al., 2009). Dechlorination by these consortia grown in stainless alloy vessels seems comparable to pure cultures of *Dehalococcoide*s grown in glass vessels, and the fact that no description of the unstable activity in the stainless alloy vessels was reported suggests two possibilities: the strain NIT01 has a particular sensitivity to the stainless alloy, or the other bacteria in the consortia increase the inhibitory effect on *Dehalococcoides* by this alloy.

Among the cultures that showed successful dechlorination of 1 mM TCE to ETH, the cell density of the strain NIT01 was 6.4 × 10^7^ and 1.1–15 × 10^8^ cells /mL in the glass and stainless alloy vessels, respectively. The biomass yield of NIT01 was 3.0 × 10^7^ cells/μmol‐released Cl^−^ in the glass vessels (*R* ^2^ = 0.95) and 2.4 × 10^7^ cells/μmol‐released Cl^−^ in the stainless alloy vessels (*R* ^2^ = 0.0082). The yield of *Dehalococcoides* is discussed more in Section 3.5.

### Effect of solid metal pieces on dechlorination by *D. mccartyi* strain NIT01

**FIGURE 2:**
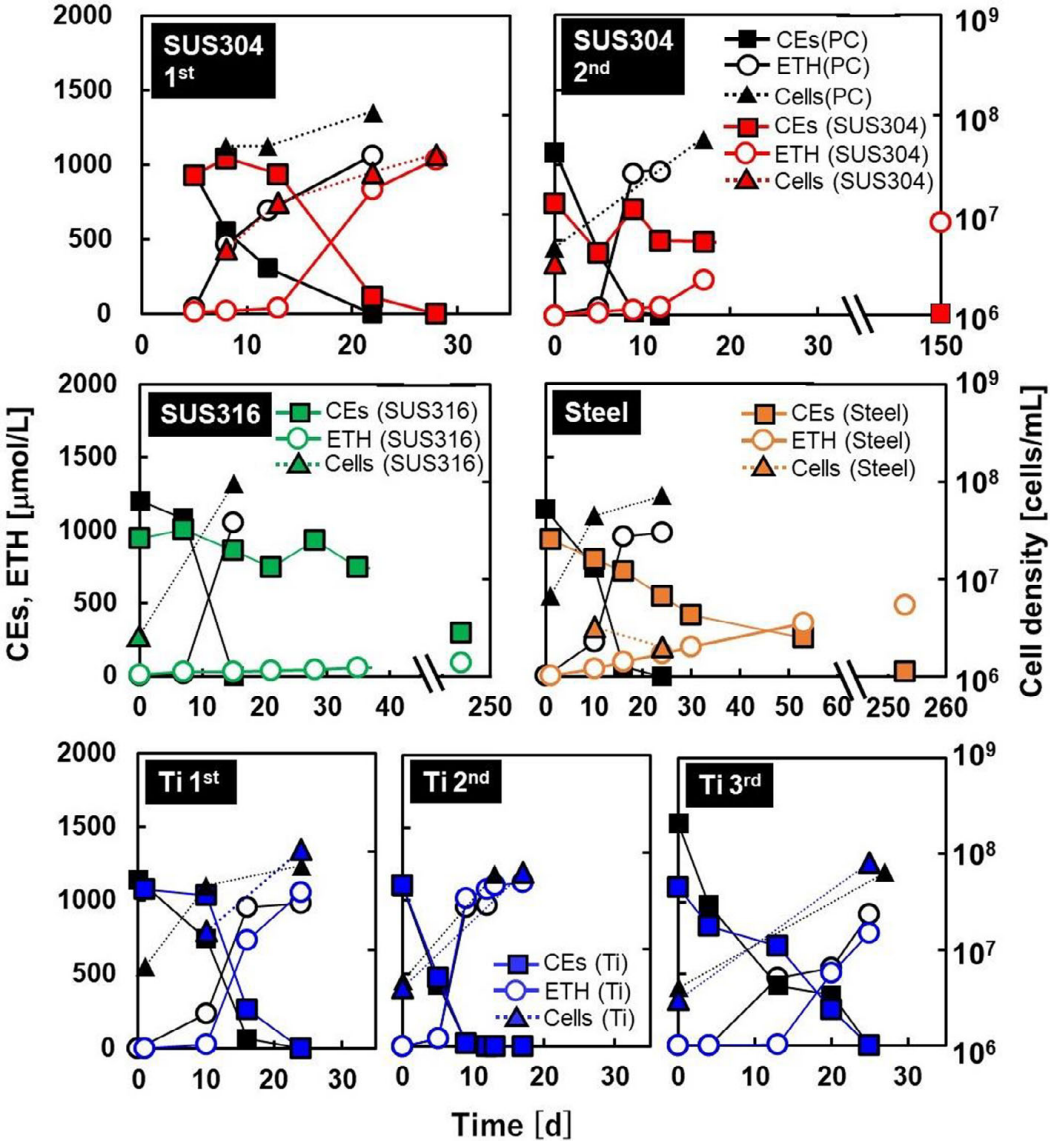
Effect of solid metal pieces on dechlorination of TCE to ETH and growth of *Dehalococcoides mccartyi* strain NIT01. The data shown are the average of duplicate readings of each culture. However, SUS304 1st and SUS316 at Day 244, and steel at Day 252 are single determinations. CEs, chloroethenes; ETH, ethene; PC, positive control; TCE, trichloroethene; Ti, titanium.

To select a metal vessel for the large‐scale culture of *D. mccartyi* strain NIT01, the strain was cultured with pieces of four different metals (SUS304, SUS316, steel, and titanium), and dechlorination by these cultures was compared with that by the culture grown without metal pieces (PC culture) (Figure 2). Two cultures were prepared for each condition, and the data in Figure 2 indicate averages of the duplicates. The *D. mccartyi* strain NIT01 dechlorinated 95% of 1 mM TCE to ETH within 22 days in the absence of metal pieces but required 28 days with SUS304 (Figure 2). The delay in the completion of TCE‐to‐ETH dechlorination was more apparent in the second batch of *D. mccartyi* strain NIT01 culture that was grown with used SUS304 pieces, that is, the pieces taken from the first culture. The second PC culture dechlorinated 95% of 1 mM TCE to ETH within 9 days, but at 17 days of the second culture with SUS304 pieces, 492 μmol/L CEs remained. SUS316 drastically inhibited dechlorination and allowed *D. mccartyi* strain NIT01 to dechlorinate only 9.1% of the spiked TCE even after 244 days. Steel showed a stronger inhibition of dechlorination than SUS304 but weaker than that shown by SUS316. In contrast, the titanium pieces did not inhibit dechlorination, and the reused titanium pieces always showed dechlorination similar to that shown by the PC culture (Figure 2). The cell density in the PC cultures that completely dechlorinated 1 mM TCE to ETH was in the range of 5.7–15 × 10^7^ cells/mL. The cell density values for the first culture grown with SUS304 and the three cultures grown with titanium were 4.1 × 10^7^ cells/mL and 5.8–10 × 10^7^ cells/mL, respectively. These data suggested that stainless alloy inhibited dechlorination and decreased growth yield.

SUS316 and SUS304 are the most popular stainless alloys and are widely used in applications that require high corrosion resistance, which is provided by a high chromium content (El‐Eskandrany & Al‐Azmi, 2016). In contrast, stainless alloys have shown no antibacterial activity in previous studies; they showed no inhibition of *Escherichia coli* growth (Jin et al., 2015; Wang et al., 2013) and resulted in less biofilm formation but a higher suspended cell density of *Legionella* (Rogers et al., 1994) compared with that of plastics, and there were no differences in biofilm formation in drinking water between stainless alloys and plastic surfaces (Zacheus et al., 2000). On the other hand, stainless alloys are known to be degraded by acid and high temperatures (Islam et al., 2018) and corroded under high salt (Islam et al., 2018) or reducing conditions (Islam et al., 2018). Corrosion is also sometimes facilitated in the presence of biomolecules, such as proteins (Hedberg et al., 2013) or microorganisms (Jin et al., 2019). These reports suggest that the inhibition of dechlorination by metal pieces might be caused by the ions released from the metals during autoclaving, or long incubation under reductive conditions.

### Changes in the surface of the solid metals

**FIGURE 3:**
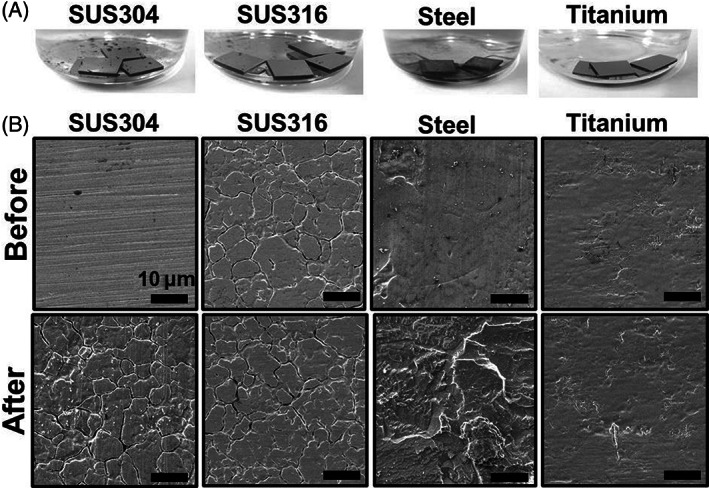
Metal pieces in cultures and changes in surface morphology. Panels (A,B) indicate the apparatus of metal pieces in the cultures (A) and changes in the surface morphology of metal pieces before and after culture (B). SUS304 was used twice with the same piece of metal and was incubated for 28 days (first time) and 150 days (second time) for a total of 178 days. SUS316 and steel were incubated once each and incubated for 183 and 192 days, respectively. The same piece of titanium was used in triplicate and incubated for a total of 101 days: 35 days the first time, 17 days the second time, and 49 days the third time.

SEM images showed changes in the surface of SUS304 and steel pieces after incubation with NIT01, whereas no change was observed in the surface of titanium pieces (Figure 3). The surface of SUS304 was relatively smooth and cracked after incubation with NIT01. The surface of the steel was uneven and became more so after culturing with strain NIT01. The crack was also observed on the surface of the steel pieces after 28 days of incubation without NIT01 (Figure S2), suggesting that the elution of metal ions from the steel pieces under the reduced condition occurred regardless of bacterial inoculation. SUS304 without bacterial inoculation maintained a smooth surface after 28 days of incubation (Figure S2), although, due to differences in the total incubation times and the number of autoclaving, it could not be determined if the crack in the culture was facilitated by bacterial inoculation. The surface of SUS316 was originally rough; therefore, it was difficult to visually check the apparent change before and after incubation.

To determine whether metal ions were released from the stainless alloy and steel pieces, we measured the total iron ion concentration, that is, Fe^2+^ and Fe^3+^, in the cultures with and without the metal pieces. The concentration of total iron ions in the medium was 9.2 μM, which slightly decreased to 8.5 μM in the PC culture, suggesting the incorporation of iron ions into cell biomass. However, the total iron ion concentration increased in the cultures with SUS304, SUS316, and steel pieces, although the concentrations varied among the samples (Table S1). At the highest concentration, the total iron concentration reached 235 μM with SUS304 at 36 days, 276 μM with SUS316 at 244 days, and 1000 μM with steel at 252 days. The major ion was Fe^2+^, and Fe^3+^ was <14% of the total iron ions in the cultures with SUS304 and SUS316. These results indicated that the iron ion was released from SUS304, SUS316, and steel in the culture with strain NIT01.

Upon XRF analysis, only the culture with SUS316 showed an apparent higher peak of Fe (6.404 and 7.058) in the salts precipitated by drying the liquid culture, along with a possible minor presence of Cr (5.415 and 5.947) (Figure S3). All other metals in the stainless alloys were not detected at proper relative intensities in all three cultures.

### Effect of metal ions on dechlorination by *D. mccartyi* strain NIT01

The release of Fe^2+^ from the metal pieces motivated us to evaluate the effects of Fe^2+^, Ni^2+^, and Cr^3+^—which were main components of the stainless alloys (>8%) and possibly released from the stainless alloy—on dechlorination by *D. mccartyi* strain NIT01. The culture supplemented with Fe^2+^, Ni^2+^, and Cr^3+^ indicated different sensitivities of the *D. mccartyi* strain NIT01 to the metal ions.

**FIGURE 4:**
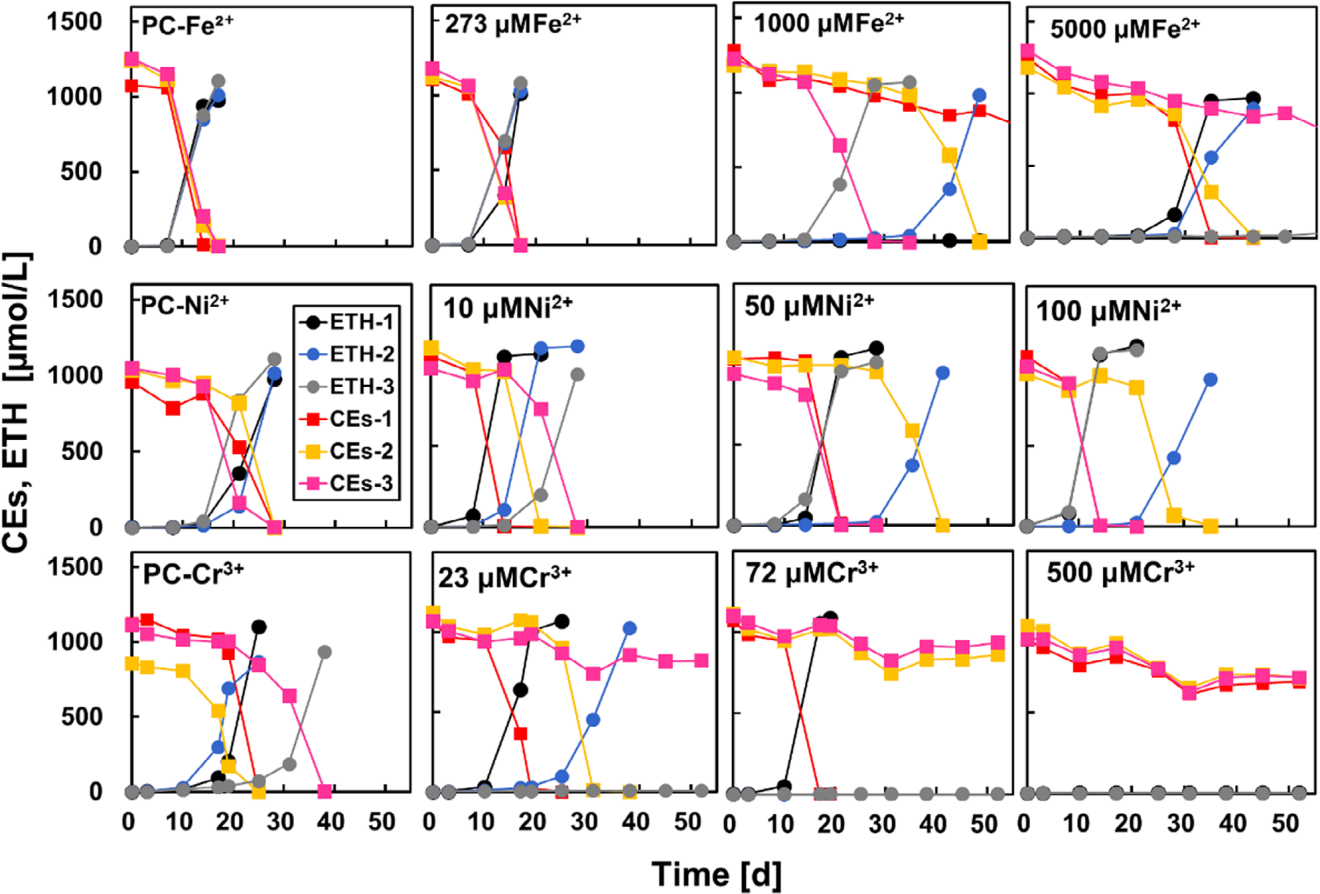
Effect of metal ions on the dechlorination by *Dehalococcoides mccartyi* strain NIT01. Metal ions were added to the cultures at the following concentrations: 273, 1000, and 5000 μM Fe^2+^; 10, 50, and 100 μM Ni^2+^; and 23, 72, and 500 μM Cr^3+^. CE, chloroethenes; ETH, ethene; PC, positive control. The three cultures were prepared in similar conditions, and all the data are presented based on a single determination from each culture.

The cultures supplemented with Fe^2+^ at concentrations of ≤273 μM showed dechlorination comparable to that shown by PC (Figure 4). However, the dechlorination was delayed and required ~2–6.5 times longer incubation periods for completion with ≥1000 μM. Ni^2+^ did not inhibit dechlorination at ≤100 μM. In the culture with SUS 304 or SUS 316, the Ni^2+^ concentration was likely <100 μM because the highest eluted iron concentration in SUS304 and SUS316 in this study was 276 μM, and the relative abundance of Ni is 8%–14% for the stainless alloy. However, the elution of metal ions from the stainless alloys was reported to vary depending on the alloy, ion conductivity, temperature, and redox conditions (Hedberg et al., 2013; Islam et al., 2018). The *D. mccartyi* strain NIT01 was most sensitive to Cr^3+^, but the dechlorination activity varied among the three cultures. Cr^3+^ inactivated one of the three cultures at a concentration of 23 μM but showed no effect on the other two. Cr^3+^ at a concentration of 72 μM inactivated two cultures, and at a concentration of 500 μM inactivated all three cultures. Therefore, the increase in Cr^3+^ concentration resulted in an increased number of inactive cultures.

The finding that *D. mccartyi* strain NIT01 showed the highest sensitivity to Cr^3+^ suggested that the minor concentration of Cr in the SUS316 culture liquid phase, along with the eluted Fe^2+^ from SUS304 and SUS316, was too low to inhibit dechlorination. It also suggested that Cr^3+^ inhibited dechlorination with the relatively higher Cr concentrations in SUS304 and SUS316. Cr^3+^ has also been shown to inhibit PCE dechlorination by *D. mccartyi* strain CG1 at a concentration of 38 μM and considerably suppress dechlorination at a concentration of 192 μM (Lu et al., 2020). In cultures grown with steel, Fe^2+^ was eluted from steel pieces at a concentration comparable to that, which inhibited dechlorination by *D. mccartyi* strain NIT01. Thus, the inhibition of TCE dechlorination by steel pieces was concluded to be caused by eluted Fe^2+^. This agreed well with a previous study that revealed that 0.54 mM Fe^2+^ did not inhibit PCE dechlorination by a consortium containing *Dehalococcoides* (Yoshikawa et al., 2021), whereas 1.75 mM Fe^2+^ inhibited TCE dechlorination by KB‐1 culture (Paul & Smolders, 2014). It is also believed that Ni^2+^ does not inhibit dechlorination because *D. mccartyi* strain NIT01 has less sensitivity towards Ni^2+^. However, Ni^2+^ has been reported to reversibly inhibit the Ni‐Fe hydrogenase activity of the membrane and soluble fractions of *D. mccartyi* strain CBDB1 (Jayachandran et al., 2004) at 75 μM, although the effect of Ni^2+^ on the whole‐cell was not described in that study and has never been investigated to the best of our knowledge. The possible mechanism underlying the inhibition of dechlorination by Cr^3+^ has been reported to be genotoxic effects and alternation of the enzyme structure (Cervantes et al., 2001).

### Large‐scale culture of *D. mccartyi* strain NIT01 using titanium vessel

**FIGURE 5:**
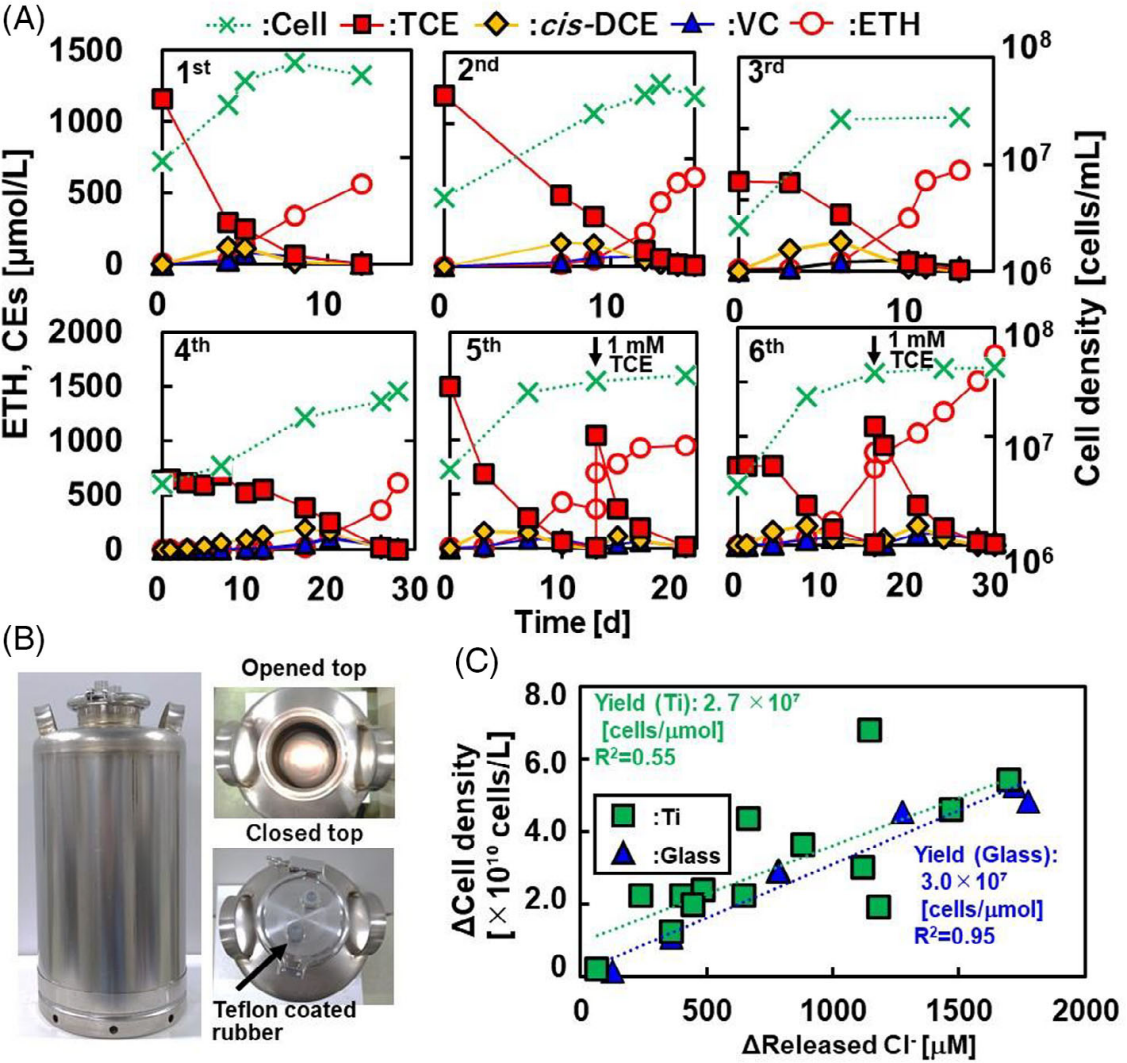
Dechlorination of TCE by *Dehalococcoides mccartyi* strain NIT01 and its growth in the titanium vessel. Panels (A–C) show the six‐batches result of TCE dechlorination by strain and growth (A), the apparatus of the titanium vessel (B), and the growth yield of *D. mccartyi* strain NIT01 (C), respectively. The first to fifth batches were cultured at an inoculation rate of 16% and the sixth batch was cultured at an inoculation rate of 5%. The cell densities of the first, third, fourth, fifth, and sixth are presented as the average of duplicate experiments, whereas the other data are of single determinations. *cis*‐DCE, *cis*‐1,2‐dichloroethene; ETH, ethene; TCE, trichloroethene; VC, vinyl chloride.

Because titanium showed no inhibition of dechlorination by *D. mccartyi* strain NIT01, 10 L‐scale culturing of this strain was performed using a titanium vessel used for heating Japanese traditional alcohol (Figure 5B). Five batches, the first to fifth, with an inoculation rate of 16% were prepared using the same vessel repeatedly, and all trials stably dechlorinated 1 mM TCE to ETH within incubation periods of 12–28 days, with cell densities reaching 2.8–7.7 × 10^7^ cells/mL (Figure 5A). Moreover, a 6th batch was prepared with a decreased inoculation rate of 5%, and the culture dechlorinated 1 mM TCE to ETH within 16 days, with cell densities reaching 3.9 × 10^7^ cells/mL (Figure 5A). The cell densities at Cl^−^ released at >1800 μM varied, and additional spiking of TCE did not linearly increase the cell density in the fifth and sixth batches; the variation was probably caused by non‐linear death, such as cell lysis caused by phages (Waller et al., 2012).

The cell density of the strain NIT01 cultured with 1 mM TCE using a titanium vessel was 4.2 ± 1.5 × 10^7^ cells/mL; this value was lower than the cell densities of other pure *Dehalococcoides* strains 195, FL2, and MB (7.8–8.9 × 10^7^ cells/mL), with lower concentrations of TCE (315–550 μM) (Cheng & He, 2009; He et al., 2005) (Table S2).

The growth yield in the titanium vessel was 2.7 × 10^7^ cells/μmol released Cl^−^ and was similar to that in the glass vessel (3.0 × 10^7^ cells/μmol released Cl^−^) (Figure 5C) when only the data for Cl^−^ released at <1800 μM were used. The yield was relatively lower than that reported for other *Dehalococcoides* strains, FL2 and MB, which were cultured with TCE (7.8 and 8.6 × 10^7^ 16S rRNA gene copies/μmol released Cl^−^, respectively) (Cheng & He, 2009; He et al., 2005) (Table S2). The reason for the big gap in yields between the different strains is unknown. The yield seemed to increase when *Dehalococcoides* strains were cultured with TCE periodically added at low concentrations; the yield of strain CBDB1 was 13 × 10^7^ cells/μmol released Cl^−^ when cultured with spiked 50 μM TCE many times (Marco‐Urrea et al., 2011). The yield also varied with different CEs; in *Dehalococcoides* strains FL2, VS, and GT that were cultured with VC, the yield amounted to 17–51 × 10^7^ 16S rRNA gene copies/μmol released Cl^−^, whereas strain FL2 showed a lower yield of 7.8–8.4 × 10^7^ 16S rRNA gene copies/μmol released Cl^−^ with TCE or DCE (Cupples et al., 2003; He et al., 2005; Sung et al., 2006). The aromatic and heterocyclic organohalides were well investigated in strain CBDB1 and ranged from 0.9–51 × 10^7^ cells/μmol released Cl^−^ (Cooper et al., 2015) to 12 × 10^7^ cells/μmol released Br^−^ in a stirring tank reactor continuously fed with brominated tyrosine (Reino et al., 2023).

## CONCLUSIONS

This study demonstrated the growth of *D. mccartyi* strain NIT01 at 10 L‐scale with at least a 5% inoculation rate in a titanium vessel and was stably reproduced even when using the same vessel multiple times. In contrast, the conventional SUS304 vessel did not grow *D. mccartyi* strain NIT01 reproducibly; this was possibly caused by eluted metal ions from the vessel when used multiple times. Our findings of how metal solids affect anaerobic microbial activity indicate that the type of metal vessel is important in regard to stable growth at large‐scale cultivation.

## AUTHOR CONTRIBUTIONS

**Masaki Asai:** Investigation (equal); writing—original draft (equal). **Yuuki Morita:** Investigation (equal). **Lingyu Meng:** Writing—review and editing (supporting). **Hidetoshi Miyazaki:** Investigation (supporting). **Naoko Yoshida:** Conceptualization (lead); funding acquisition (lead); methodology (lead); supervision (lead); writing—review and editing (lead).

## CONFLICT OF INTEREST STATEMENT

Naoko Yoshida has a patent pending for the vessel. The remaining authors have no conflicts of interest to declare.

## Supporting information


**FIGURE S1.** The photo showing the inoculation in a titanium vessel.
**FIGURE S2.** Metal pieces in medium without cells and changes in the surface morphology.
**FIGURE S3.** The x‐ray fluorescence analysis of salt precipitated from liquid cultures with and without pieces of SUS304 or SUS316.
**TABLE S1.** The concentrations of Fe^2+^ and Fe^3+^ in the cultures.
**TABLE S2.** The cell density and yield of *Dehalococcoides* strains cultures with chloroethenes.

## Data Availability

All data used in this study are available online.
