# Supplementary material for: Dehalococcoides mccartyi strain NIT01 grows more stably in vessels made of pure titanium rather than the stainless alloy SUS304
Source: Environ Microbiol Rep. 2023 Aug 18;15(6):557–67. doi: 10.1111/1758-2229.13192 (PMC10667658; doi:10.1111/1758-2229.13192)
Supplement: Supplementary file 1 — FIGURE S1. The photo showing the inoculation in a titanium vessel. FIGURE S2. Metal pieces in medium without cells and changes in the surface morphology. FIGURE S3. The x‐ray fluorescence analysis of salt precipitated from liquid cultures with and without pieces of SUS304 or SUS316. TABLE S1. The concentrations of Fe2+ and Fe3+ in the cultures. TABLE S2. The cell density and yield of Dehalococcoides strains cultures with chloroethenes. [file EMI4-15-557-s001.docx]

***Dehalococcoides mccartyi* strain NIT01 grows more stably in vessels made of pure titanium rather than the stainless alloy SUS304**

*DEHALOCOCCOIDES MCCARTYI* STRAIN NIT01 GROWS

Masaki Asai^1^, Yuki Morita^1^, Lingyu Meng^1^, Hidetoshi Miyazaki^2^, Naoko Yoshida^1,*^

^1^Department of Civil Engineering, Nagoya Institute of Technology, Nagoya, Japan

^2^Department of Physical Science and Engineering, Nagoya Institute of Technology, Nagoya, Japan

Masaki Asai and Yuki Morita contributed equally to this work.

**^*^Corresponding author:** Naoko Yoshida

Department of Civil Engineering, Nagoya Institute of Technology, Nagoya, Aichi 466 0061,

E-mail: [yoshida.naoko@nitech.ac.jp](mailto:yoshida.naoko@nitech.ac.jp)

**FIGURE S1. The photo showing the inoculation in a titanium vessel.**

**FIGURE S2. Metal pieces in medium without cells and changes in the surface morphology.**

**FIGURE S3. The X-ray fluorescence analysis of salt precipitated from liquid cultures with and without pieces of SUS304 or SUS316.**

**TABLE S1. The concentrations of Fe^2+^ and Fe^3+^ in the cultures.**

**TABLE S2. The cell density and yield of *Dehalococcoides* strains cultures with chloroethenes.**


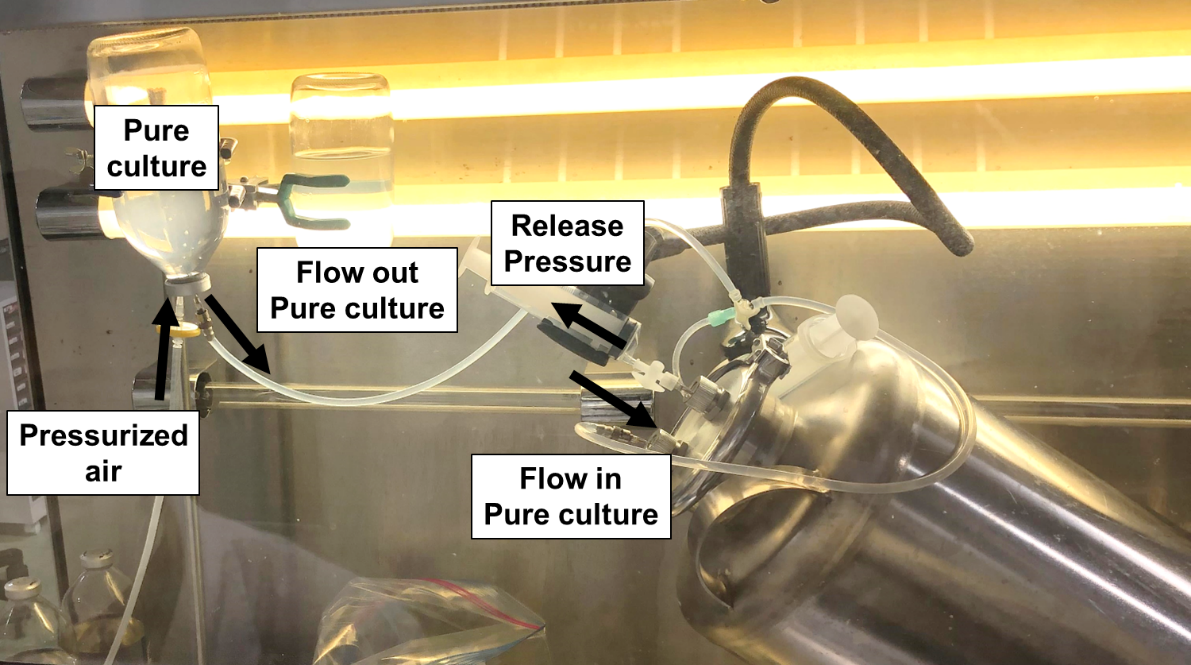


**FIGURE S1. The photo showing the inoculation in a titanium vessel.**


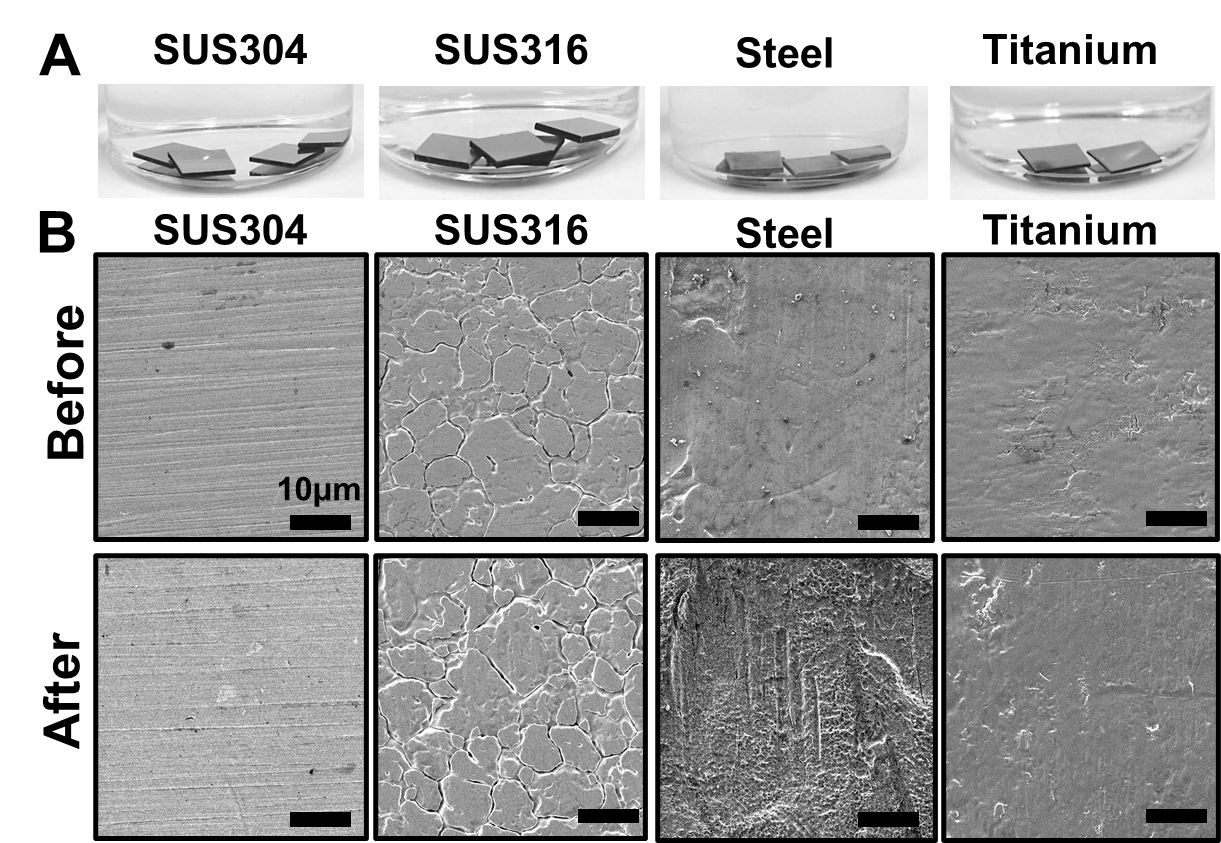


**FIGURE S2. Metal pieces in medium without cells and changes in the surface morphology.** Panels (A, B) show the apparatus of metal pieces in media without NIT01 (A) and changes in the surface morphology of metal pieces before and after incubation (B). All metal pieces were incubated for 28 days.


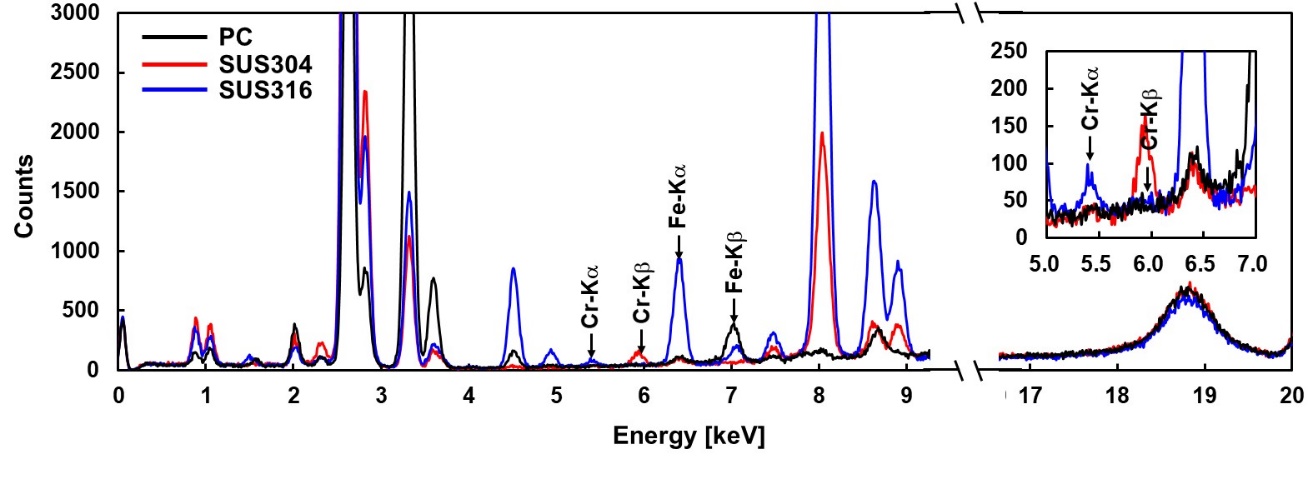
**FIGURE S3. The X-ray fluorescence analysis of salt precipitated from liquid cultures with and without pieces of SUS304 or SUS316.** The insert is an enlarged image focusing on the energy range from 5.0 to 7.0 keV.

**TABLE S1. The concentrations of Fe^2+^ and Fe^3+^ in the cultures.**

| Culture | Total iron ion concentration  (µM) | Fe^2+^ (µM) | Fe^3+^ (µM) | Incubation period (d) |
| --- | --- | --- | --- | --- |
| Medium* | 9.2 | - | - | - |
| PC culture -1* | 8.5 | - | - | 24 |
| PC culture -2 | 18 | 18 | 0 | 36 |
| SUS304 culture-1 | 235 | 207 | 28 | 36 |
| SUS304 culture-2* | 76 | - | - | 150 |
| SUS316 culture-1 | 183 | 158 | 25 | 36 |
| SUS316 culture-2 | 276 | - | - | 244 |
| Steel culture | 1000 | - | - | 252 |

PC culture, culture grown without metal pieces, and d, number of days. All data are presented as average values of the duplicate analyses, except for the data marked with *, which were determined by a single measurement.

**TABLE S2. The cell density and yield of *Dehalococcoides* strains cultures with chloroethenes.**

| Strain | e^−^ acceptor | Volume (ml) | Cell density (10^7^ cells/mL) | Yield (10^7^ Cells/µmol Cl^−^) or (10^7^ copies/µmol Cl^−^) | Method | Inoculum  rate (%, v/v) | Time (d) | Reference |
| --- | --- | --- | --- | --- | --- | --- | --- | --- |
| 195 | 50 µmol/bottle TCE (500 µmol/L-culture TCE)^*1^ | 100 | 9.5 | -  (9.3)*^2^ | qPCR targeting *tceA* | 2 | (45)^*3^ | (He *et al.*, 2007) |
| CBDB1 | 50µM TCE was repeatedly spiked | 30 | - | (13)^*4^ | direct microscopic cell counts | 30 | (10)^*5^ | (Marco-Urrea *et al.*, 2011) |
| BAV1 | 80 µmol VC /bottle | 100 | 5.0 ± 0.26 | (6.2)^*6^ | qPCR targeting 16S rRNA gene | - | - | (He *et al.*, 2003) |
| FL2 | 37.2 µmol TCE /bottle  (372 µmol/L-culture TCE) *^1^ | 100 | 4.7 ± 0.5 | 7.8 ± 0.9 | qPCR targeting 16S rRNA gene | 2 | - | (He *et al.*, 2005) |
| FL2 | 31.5 µmol *cis*-DCE /bottle  (315 µmol/L-culture *cis*-DCE) *^1^ | 100 | 2.2 ± 0.2 | 8.4 ± 0.8 | qPCR targeting 16S rRNA gene | 2 | - | (He *et al.*, 2005) |
| FL2 | 30.1 µmol *trans*-DCE /bottle  (301 µmol/L-culture *trans*-DCE)*^1^ | 100 | 2.1 ± 0.4 | 8.1 ± 1.5 | qPCR targeting 16S rRNA gene | 2 | - | (He *et al.*, 2005) |
| FL2 | 0.52 mM VC | 100 | 13 ± 1.3 | 17 ± 2.6 | qPCR targeting *tceA* | 3 | 84 | (Yan *et al.*, 2021) |
| GT | 0.32 mM TCE | 100 | - | (31)^*7^ | qPCR targeting 16S rRNA gene | 0.5 | (33)^*3^ | (Sung *et al.*, 2006) |
| GT | 0.55 mM VC | 100 | 9.7 ± 0.12 | 25 ± 1.3 | qPCR targeting 16S rRNA gene | 0.5 | (20)^*3^ | (Sung *et al.*, 2006) |
| MB | 0.55 µmol/mL TCE  (550 µmol/L-culture TCE)*^1^ | 100 | 3.1 ± 0.07 | 8.6 ± 0.13 | qPCR targeting 16S rRNA gene | 1–2 | (7)^*3^ | (Cheng and He, 2009) |
| CG1 | 0.7 mM PCE | 10 | 13 | 12 | qPCR targeting 16S rRNA gene | - | (60)^*3^ | (Wang *et al.*, 2014) |
| VS*^8^ | 130 µL VC /bottle and 250 µL VC /bottle spiked at 15 d  (5.8 µmol VC /bottle and 12 µmol VC /bottle spiked at 15 d) | 60 | 1.2 ± 0.4 | 51 | qPCR targeting 16S rRNA gene | (1.7)^*9^ | (19)^*3^ | (Cupples *et al.*, 2003) |
| KB-1 | 30 μeeq TCE /bottle  (250 µmol/L-culture TCE) ^*1^ | 20 | - | 36 ± 13 | qPCR targeting 16S rRNA gene | 5 | - | (Duhamel *et al.*, 2004) |

TCE, trichloroethene; DCE, dichloroethene; ETH, ethene; VC, vinyl chloride; qPCR, quantitative-polymerase chain reaction; and d, number of days.

^*1^ Calculated value from the described concentration and culture volume in the paper

^*2^ Calculated value from the data approximately read from a graph; VC and ETH concentrations were assumed 20 μmol/bottle.

^*3^ The value was approximately read from a graph.

^* 4^ The data was updated by the author’s correction.

^*5^ The value is the time needed to dechlorinate 45 μM TCE and was approximately read from a graph.

^*6^ Calculated value of 16SrRNA gene copies per the dechlorinated VC, approximately read from a graph. 12 ×10^9^ copies were assumed to be grown with 200 μmol of VC dechlorination.

^*7^ The yield was originally calculated as the 16S rRNA gene copy numbers normalized by the produced ETH. In this table, the value was recalculated to be normalized by released Cl^−^ concentration.

^*8^ Highly enriched culture

^*9^ Calculated value from the description that 1mL of culture was inoculated in 59 mL of an anaerobic medium.

**References**

Cheng, D. and He, J. (2009) Isolation and characterization of “*Dehalococcoides*” sp. strain MB, which dechlorinates tetrachloroethene to *trans*-1,2-dichloroethene. *Appl Environ Microbiol* **75**: 5910–5918.

Cupples, A.M., Spormann, A.M., and McCarty, P.L. (2003) Erratum: Growth of a *Dehalococcoides*-like microorganism on vinyl chloride and *cis*-dichloroethene as electron acceptors as determined by competetive PCR (Applied and Environmental Microbiology (2003) 69:2 (953-959). *Appl Environ Microbiol* **69**: 4342.

Duhamel, M., Mo, K., and Edwards, E.A. (2004) Characterization of a highly enriched *Dehalococcoides*-containing culture that grows on vinyl chloride and trichloroethene. *Appl Environ Microbiol* **70**: 5538–5545.

He, J., Holmes, V.F., Lee, P.K.H., and Alvarez-Cohen, L. (2007) Influence of vitamin B_12_ and cocultures on the growth of *Dehalococcoides* isolates in defined medium. *Appl Environ Microbiol* **73**: 2847–2853.

He, J., Kirsti M. Ritalahti, Kun-Lin Yang, Stephen S. Koenigsberg, and Frank E. Löffler (2003) Detoxification of vinyl chloride to ethene coupled to growth of an anaerobic bacterium. *Nature* **424**: 62–65.

He, J., Sung, Y., Krajmalnik-Brown, R., Ritalahti, K.M., and Löffler, F.E. (2005) Isolation and characterization of *Dehalococcoides* sp. strain FL2, a trichloroethene (TCE)- and 1,2-dichloroethene-respiring anaerobe. *Environ Microbiol* **7**: 1442–1450.

Marco-Urrea, E., Nijenhuis, I., and Adrian, L. (2011) Transformation and carbon isotope fractionation of tetra-and trichloroethene to *trans*-dichloroethene by *Dehalococcoides* sp. strain CBDB1. *Environ Sci Technol* **45**: 1555–1562.

Sung, Y., Ritalahti, K.M., Apkarian, R.P., and Löffler, F.E. (2006) Quantitative PCR confirms purity of strain GT, a novel trichloroethene-to- ethene-respiring *Dehalococcoides* isolate. *Appl Environ Microbiol* **72**: 1980–1987.

Wang, S., Chng, K.R., Wilm, A., Zhao, S., Yang, K.-L., Nagarajan, N., and He, J. (2014) Genomic characterization of three unique *Dehalococcoides* that respire on persistent polychlorinated biphenyls. *Proc Natl Acad Sci* **111**: 12103–12108.

Yan, J., Wang, J., Villalobos Solis, M.I., Jin, H., Chourey, K., Li, X., et al. (2021) Respiratory vinyl chloride reductive dechlorination to ethene in TceA-expressing *Dehalococcoides mccartyi*. *Environ Sci Technol*.
